# Assessment of the Gut Microbiota during Juice Fasting with and without Inulin Supplementation: A Feasibility Study in Healthy Volunteers †

**DOI:** 10.3390/foods11223673

**Published:** 2022-11-16

**Authors:** Kerstin Thriene, Virginie Stanislas, Lena Amend, Till Strowig, Karin B. Michels

**Affiliations:** 1 Institute for Prevention and Cancer Epidemiology, Faculty of Medicine and Medical Center, University of Freiburg, 79110 Freiburg, Germany; 2 Department of Microbial Immune Regulation, Helmholtz Center for Infection Research, 38124 Braunschweig, Germany; 3 Cluster of Excellence RESIST (EXC 2155), Hannover Medical School, 30625 Hannover, Germany; 4 Department of Epidemiology, Fielding School of Public Health, University of California, Los Angeles, CA 90095, USA

**Keywords:** inulin, prebiotics, human gut microbiome, dietary intervention, fasting, juice fasting, feasibility study, pilot study, crossover study

## Abstract

Prebiotic inulin consumption provides health benefits to the host and has also been associated with a reduction in hunger cravings. We conducted a pilot crossover study to investigate the feasibility of a juice fasting intervention with and without inulin supplementation. We also examined trends of how the microbial community in the human gut adapts to juice fasting as well as to inulin intake during juice fasting. Six healthy volunteers were fasting for three consecutive days consuming a total of 300 kcal daily provided by vegetable juices, framed by two days with a total daily calorie intake of 800 kcal, respectively. During one fasting period, participants consumed additionally 24 g of inulin daily. Stool samples were collected for the analysis of the microbial composition using 16S rRNA gene sequencing. Although no significant uniform changes were observed on the microbiome, quantitative changes in the microbial composition suggest a stronger decrease in alpha-diversity after fasting compared to the fasting intervention with additional inulin intake. The intake of inulin did not affect compliance for the fasting intervention but appeared to increase relative abundance of *Bifidobacteria* in participants who tolerated it well. Further studies with a larger sample size to overcome inter-individual microbiota differences are warranted to verify our observations.

## 1. Introduction

The human gastrointestinal tract (GIT) is populated by a vast number of microorganisms (including bacteria, fungi, viruses, archaea, and protists) that are referred to as the “gut microbiota”. This microbial community varies widely between individuals, is adaptable to environmental influences and changes over the course of human life [1,2]. Numerous research reports suggest that there is a symbiotic relationship between microbiota and the host. Gut bacteria are crucial for maintaining normal host physiology by protecting against pathogens, training the immune system, assisting with nutrient uptake, and processing dietary compounds [3,4,5,6]. When symbiotic homeostasis is out of balance, gastrointestinal disorders such as intestinal bowel diseases (IBDs) [7], irritable bowel syndrome (IBS) [8], and coeliac disease [9], as well as colorectal cancer (CRC) [10,11], are more likely to occur.

Food compounds that are inaccessible for human digestive enzymes but can be metabolized by microorganisms in the colon and provide health benefits for the host are called prebiotics [12]. The consumption of prebiotics appears to influence the composition of the human gut microbiota and may, among other things, lead to an improvement in the barrier function of the gut as well as impact intestinal motility [13]. In IBDs such as Crohn’s disease (CD) and ulcerative colitis (UC), the use of pro- and prebiotics shows promising results but larger randomized controlled trials are needed for confirmation [13,14,15].

Inulin is a naturally occurring prebiotic that is produced and stored in various plants such as chicory. The intake of inulin has been suggested to modulate the composition of gut microbiota, regulate lipid metabolism, and might reduce the risk of colon cancer [16]. The fermentation of prebiotics by the gut microbiota leads to the production of metabolites such as short-chain fatty acids (SCFA) such as acetate, propionate, or butyrate, which can be absorbed by the epithelial cells in the GIT. Those enterocytes metabolize most of the butyrate directly. Large amounts of propionate are metabolized in the liver while the majority of the acetate is transported to the periphery via the blood stream. The latest research suggests that SCFA results in a wide range of health benefits including improvements in body composition, glucose homeostasis, blood lipid profiles, and reduced body weight and colon cancer risk [7,8]. Inulin is a commercially available dietary supplement that is not expected to cause serious adverse effects. However, some symptoms have been reported, although inconsistently, in different studies [17]. The reported adverse effects in studies with healthy participants were flatulence, bloating, and abdominal pain [18,19].

The composition of the microbiota changes quickly and drastically due to environmental changes induced by the host’s diet, age, lifestyle, use of drugs such as antibiotics, and disease [2,20,21,22,23]. These changes are thought to include varying degrees of reduced caloric intake that occur during some types of fasting, but few studies have considered this. In general, fasting is referred to as complete or partial abstinence from solid and/or liquid nutrition for a specific period of time [24,25,26]. In this context, different feeding regimens have been administered varying from fasting approximately 14 to 18 h daily to consuming only water for days or even up to several weeks. Besides that, caloric restriction, which is traditionally defined as a reduced daily caloric intake of 15–40%, is a promising approach to prolong life and to prevent cancer and was found to be efficacious in the treatment of hypertension, chronic pain syndromes, metabolic syndrome, and rheumatic diseases [26,27]. Conversely, long term caloric restriction can cause adverse effects such as nutrient deficiencies, cold sensitivity, anemia, menstrual irregularities, and slower wound healing [28]. Additionally, consuming a diet very low in microbiota-accessible carbohydrates can lead to starvation of the human gut microbiota, which can then lead to microbial dysbiosis [29].

In recent years, fasting has increasingly become the focus of public interest [30,31]. Juice fasting, according to Otto Buchinger, has especially become more and more popular in Germany [32,33]. With this special type of fasting, participants consume from 200 to 300 kcal daily through fruit or vegetable juices along with 2–2.5 L herbal teas and water while abstaining from other food [33]. This method is widely used in Germany and has been shown to be safe for healthy people. Sleep disturbances, fatigue, dry mouth, back pain, and hunger are among the most frequently reported side effects [34]. Although the consumption of fruit and vegetable juices seems to have some beneficial associations with human health, for example by having a positive effect on cardiovascular risk factors [35] or cognitive functions [36], there are insufficient scientific studies investigating the health benefits of juice fasting. Prebiotics such as inulin have also been associated with a decrease in hunger sensations [17,37]. They modulate the composition of the gut microbiota by providing food sources to the gut microorganisms and provide health benefits to the host via the production of metabolites such as SCFA. Including inulin in a fasting regimen might not only strengthen the positive health effects of the intervention but also support the participants by easing the side effects such as hunger sensations and nausea. To our knowledge, the effects of juice fasting in combination with inulin supplementation on the gut microbiome have not yet been studied in a human intervention study. Therefore, we aimed to implement a feasibility study to test the association between inulin consumption and the gut microbiome during juice fasting. We implemented a crossover intervention study based on a fasting protocol including vegetable juices as well as inulin during a second juice fasting period to evaluate the feasibility and evaluating crude trends on possible effects of the dietary intervention on the composition of the human gut microbiota.

## 2. Materials and Methods

### 2.1. Study Design of the Pilot Intervention Study

To assess the feasibility of a large-scale dietary intervention study investigating possible associations between juice fasting as well as inulin consumption and the composition of the human gut microbiome, six healthy participants (age: 32.6 ± 7.5 year, BMI: 22.0 ± 1.2 kg/m^2^), including four women and two men, were recruited at the University Hospital Freiburg.

The exclusion criteria included a history of chronic diseases, acute or chronic gastrointestinal symptoms (e.g., inflammatory bowel disease), one or more episodes of strong diarrhea within the past three months, fasting blood glucose levels above 100 mg/dL, blood pressure below 60/100 mmHg, pregnancy and breastfeeding, regular intake of oral pro- or prebiotic supplements or any antibiotics within the past three months, severe dietary restraints (such as veganism), plans to change the diet within the study period (besides the intervention), and smoking or consumption of more than two standard drinks of alcohol (20 g pure alcohol in total) per day.

**Figure 1:**
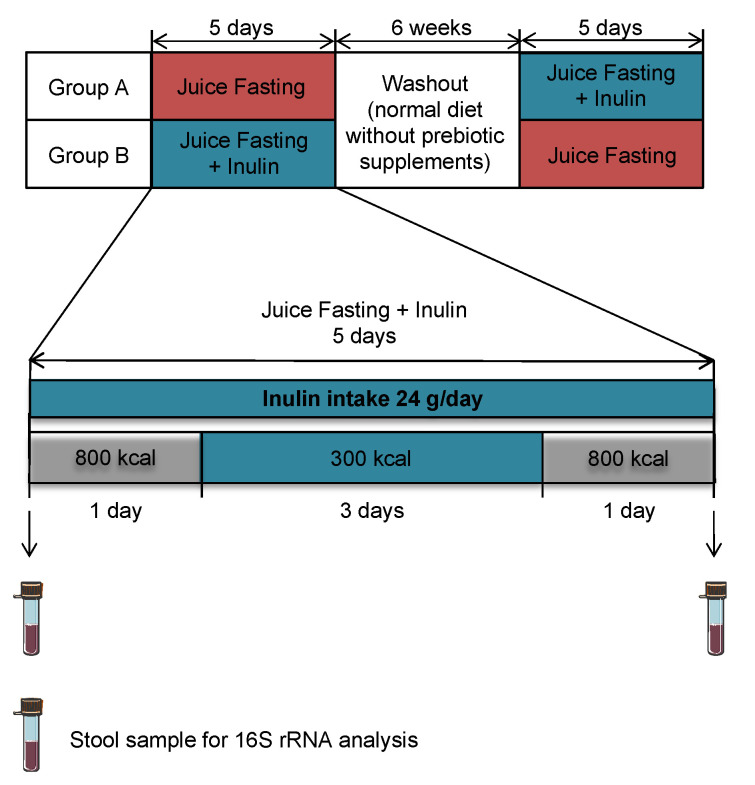
Study design of the crossover intervention. Two juice fasting interventions (one with additional 24 g inulin taken daily and one without inulin) separated by a six-week washout phase. Each fasting intervention consisted of five days. The first and the last day each included small light meals of 800 kcal in total. The three acute fasting days in the middle of the intervention include 300 kcal daily as vegetable juices and tea. Stool samples were collected right before and after each fasting intervention.

We conducted an eight-week randomized intervention trial with a crossover design that included two fasting interventions separated by a washout period to reduce the risk of carryover (Figure 1). All six participants took part in both interventions and hence acted as their own control. Within each fasting intervention participants were fasting for three consecutive days consuming a total amount of 300 kcal daily provided according to a modified fasting protocol from the Buchinger regimen [33]. During these three days, participants exclusively consumed a selection of commercially available organic vegetable juices (tomato, mixed vegetables, beetroot, sauerkraut, and carrot) provided to them. They were instructed which type of juice to consume on which day of the intervention by a diet plan (Table A1). The detailed nutrient composition of the five different juices is displayed in Table A2. The three acute fasting days were framed by two days with a total daily calorie intake of 800 kcal, respectively. During these two days, participants were instructed to consume light meals consisting mainly of vegetables and grains. Meat, fish, dairy products, sugar, alcohol, and caffeine were supposed to be excluded. The participants were provided with detailed information about suitable types and amounts of food and suitable recipes for meals. Additionally, participants were instructed to drink at least 2 L of water or unsweetened tea daily. Therefore, they were provided with a selection of tea bags containing flavors such as peppermint, rose hip, and melissa. During one of the fasting periods, participants additionally consumed 24 g of inulin daily. Vegetable juices (Alnatura Produktions-und Handels GmbH, Darmstadt, Germany), tea (Alnatura Produktions-und Handels GmbH, Darmstadt, Germany), and inulin (Aleavedis Naturprodukte GmbH, Bexbach, Germany) were provided to the participants to ensure a comparable nutrition during the intervention phases. The participants provided a food diary in which they documented if they deviated from the diet plan. The stool samples were collected in the beginning and in the end of each fasting phase for the analysis of the microbial composition using 16S rRNA gene sequencing.

This study was conducted in accordance with the Declaration of Helsinki and approved by the Ethics Committee of Albert-Ludwigs-University Freiburg (protocol code 119/19, date of approval: 4 June 2019).

### 2.2. Stool Sample Collection

The stool samples were collected by participants at home in two containers (i) native, (ii) with 96% ethanol. The participants were instructed to keep the samples in the refrigerator after collection and to return them within 24 h to the study center. The samples were immediately aliquoted and stored at −80 °C until subsequent analysis.

### 2.3. Fecal Microbial DNA Isolation

The fecal microbial DNA was extracted using a QIAamp DNA Stool Mini Kit (Qiagen, Hilden, Germany) following the manufacturer’s instructions with modifications, similar to how it was described before [38,39,40]. The temperature of the stool lysis was increased from 70 (suggested temperature in the protocol) to 95 °C 400 µL supernatant (instead of suggested volume 200 µL) was added to 15 μL of proteinase K to which 400 µL AL buffer (instead of suggested volume of 200 µL) was added followed by thorough mixing and incubation at 70 °C for 10 min. A spin column was loaded twice with 400 µL of the lysate.

### 2.4. 16S Ribosomal RNA (rRNA) Gene Sequencing

According to an established protocol previously described, 16S rRNA gene amplification of the V4 region (F515/R806) was performed [41]. Briefly, DNA was normalized to 25 ng/µL and used as an input for PCR with unique 12-base Golary barcodes incorporated via specific primers (obtained from Sigma, Saint Louis, MI, USA). The PCR was performed using Q5 polymerase (New England Biolabs, Ipswich, MA, USA) in triplicates for each sample, using PCR conditions of initial denaturation for 30 s at 98 °C, followed by 25 cycles (10 s at 98 °C, 20 s at 55 °C, and 20 s at 72 °C). After pooling and normalization to 10 nM, the PCR amplicons were sequenced on an Illumina MiSeq platform via 250 bp paired-end sequencing (PE250).

### 2.5. Bioinformatics and Statistical Analysis

Microbiome data were analyzed using Usearch11.0.667 software package (http://www.drive5.com/usearch/ (accessed on 27 May 2020)) to assemble, filter, and cluster resulting reads. The sequences were filtered for low quality reads and binned based on sample-specific barcodes using QIIME v1.8.0 [42]. The merging was performed using fastq_mergepairs with fastq_maxdiffs 30. The quality filtering was conducted using fastq_filter (fastq_maxee 1), using a minimum read length of 250 bp and a minimum number of reads per sample = 1000. The reads were clustered into 97% ID OTUs by open-reference OTU picking and representative sequences were determined by the use of the UPARSE algorithm [43]. The abundance filtering (OTUs cluster > 0.5%) and taxonomic classification were performed using the RDP Classifier executed at 80% bootstrap confidence cut off [44]. The sequences without matching reference dataset were assembled as de novo using UCLUST. The phylogenetic relationships between OTUs were determined using FastTree to the PyNAST alignment [45]. The resulting OTU absolute abundance table and mapping file were used for statistical analyses and data visualization in the R statistical programming environment package phyloseq [46]. The alpha diversity (richness and evenness) was estimated using the observed richness (number of different species directly observed in each sample) and the Shannon diversity index on the observed count values. For the following analysis, low abundant OTUs (corresponding to less than 0.5% of the abundance for all samples) were filtered out and the OTU table was converted into relative abundance. The beta diversity was estimated using Bray–Curtis distances and visualized using Non-Metric Multidimensional Scaling (NMDS). The community composition was examined at the genus-level. The species belonging to the same genus were aggregated together while the OTUs with no taxonomic annotation were categorized as “unknown”. The genus with total relative abundance over all samples inferior at 10% were aggregated into the category “Other”. Because this is a feasibility study with a small number of participants, we focus on descriptive analyses and have deliberately refrained from significance testing.

## 3. Results

### 3.1. Assessment of the Feasibility of the Study Design

Six healthy volunteers participated in this feasibility study. The compliance was complete as all six participants completed both interventions. In total, 100% of the stool samples as well as food and symptoms diaries were returned. According to the food diaries, compliance to the study protocol was high.

The reported side effects from fasting were fatigue, headache, hunger, circulatory problems, and nausea. One participant reported that these side effects severely affected his daily life during the acute fasting phase, while three participants reported moderate side effects and two participants reported almost no side effects from the fasting intervention (Table 1). Three participants stated they would fast again for three days independent of the study, while two participants would not. Four participants indicated that they could imagine trying a five-day fasting intervention, while one participant could not. All six participants indicated that they would prefer a fasting intervention with vegetable broth instead of vegetable juices. The majority stated that they did not feel the need to consume the entire 300 kcal and reported they disliked the taste of some juices. Of the five vegetable juices offered (tomato, mixed vegetable, beet, sauerkraut, and carrot juices), tomato and mixed vegetable juices were most preferred, while beetroot, sauerkraut, and carrot juices were disliked by some of the participants. Three participants stated that they experienced no side effects from inulin use and would consume inulin regularly, while three participants experienced bloating, flatulence, diarrhea, and nausea that mildly to moderately affected their daily lives.

### 3.2. Possible Associations between Inulin Consumption during Juice Fasting and the Human Gut Microbiome Composition

**Figure 2:**
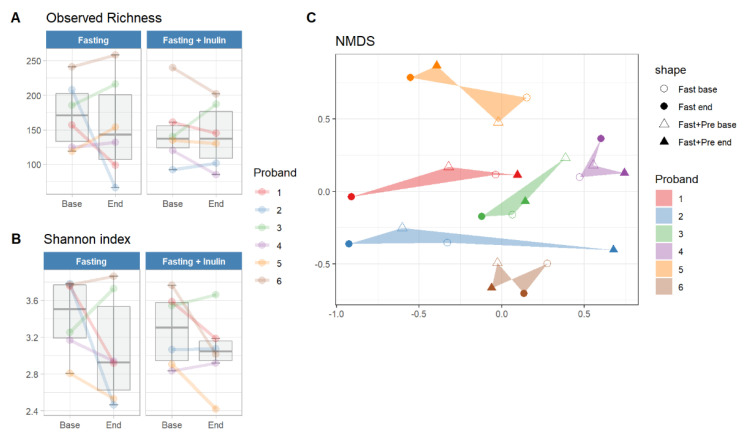
Changes in human gut microbial composition after fasting intervention and inulin intake. (**A**,**B**) Alpha diversity measures before and after fasting intervention. The lines and dots are colored according to the identities of the participants and show the individual changes in the gut microbiome community. (**C**) Beta diversity of bacterial samples based on Bray-Curtis distances and visualized using Non-Metric Multidimensional Scaling (NMDS). Colors correspond to participant identities and shapes to intervention phases.

Neither fasting intervention resulted in a significant change in microbial richness or evenness of the participants’ gut microbial composition. Nevertheless, a decrease in mean values was observed between baseline measurements and post-fasting interventions (from 160.25 (48.67) to 148.00 (57.99) for observed richness and from 3.35 (0.39) to 3.06 (0.48) for the Shannon index). The decrease was smaller for the fasting intervention that included additional inulin intake (Figure 2A,B). The ordination plot (Figure 2C) depicts the individuality of the microbial profiles, with points clustered primarily by sample identity rather than by fasting intervention or time point. The participants 2, 4, and 5 display a greater microbial change between baseline and intervention than between their two baseline samples, which may reflect a possible impact of the fasting intervention on the gut microbial composition.

**Figure 3:**
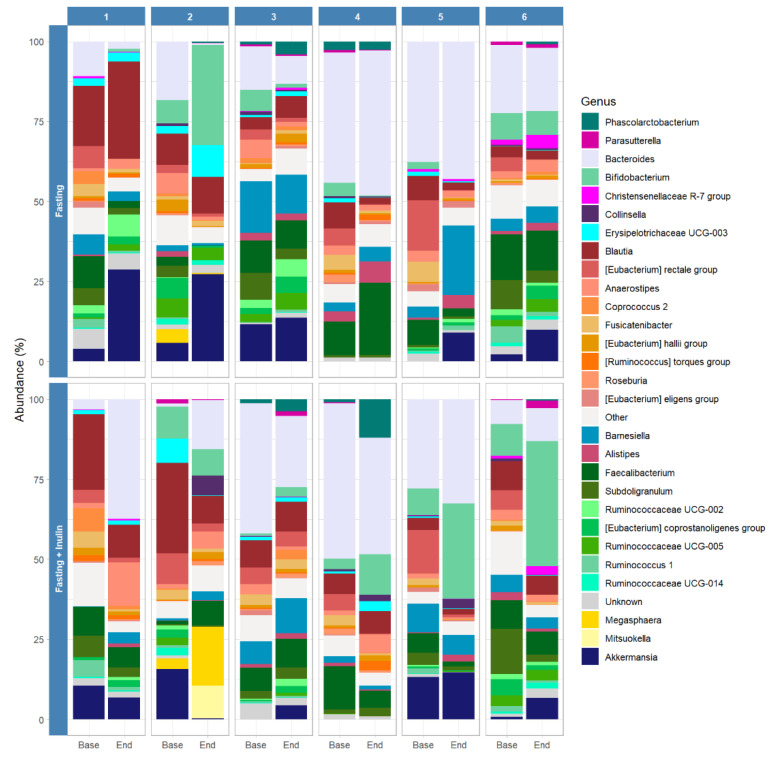
Relative abundance of the most abundant genera at the individual level before and after fasting interventions and inulin consumption. Genera from the same family are grouped next to each other by decreasing levels of abundance and colored with a similar shade of color.

**Figure 4:**
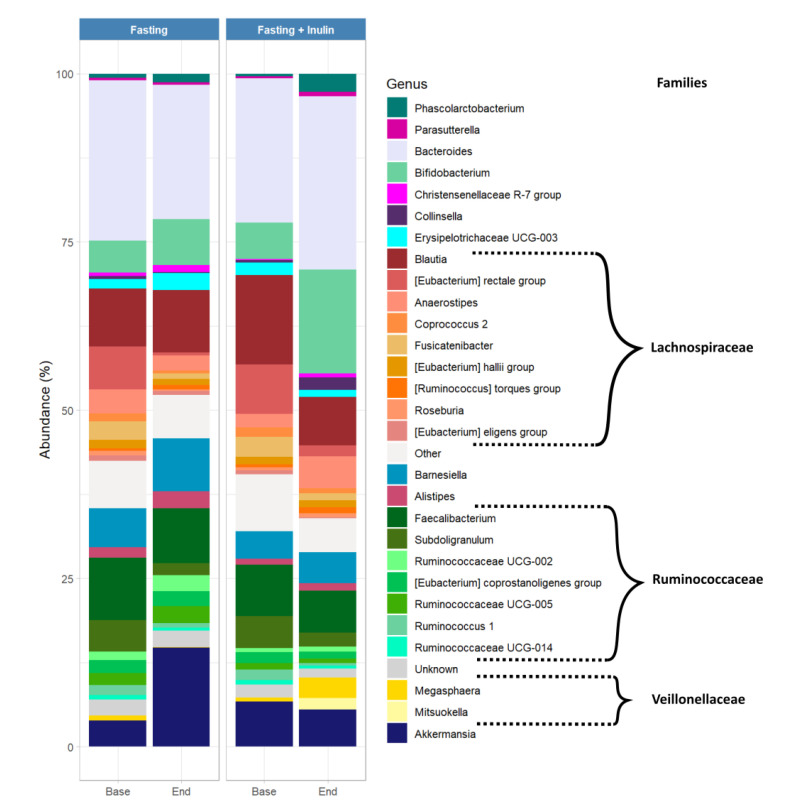
Bacterial composition of aggregated gut microbial samples before and after fasting interventions and inulin consumption. Relative abundance of the most abundant genera. Genera from the same family are grouped next to each other by decreasing level of abundance and colored with a similar shade. Names of the corresponding families are indicated by brackets.

Figure 3 depicts the strong heterogeneity and individuality of the microbial composition in our study population, with some genera observed in only one participant. Interestingly, the microbiota of participant 2 harbors genera that were not detected in the stool samples of the other participants, such as *Megasphaera* as well as *Mitsuokella*. Both genera increased especially after the fasting intervention with additional inulin. However, our analysis also displays microbial changes common to all participants (Figure 3 and Figure 4), with a visible decrease in the relative abundance of the *Eubacterium rectale group* as well as *Fusicatenibacter* after both interventions. We also observed increases in relative abundance for *Akkermansia,* especially after the fasting-only intervention. The relative abundance of the genus *Bifidobacterium* increased, particularly after the fasting intervention with supplemental inulin, and the increase was even more pronounced among participants who reported tolerating inulin well, such as participants 5 and 6.

## 4. Discussion

In this pilot human intervention study, the feasibility of a study design including two three-day fasting interventions, one with and one without additional inulin intake, was investigated. In addition, crude trends of a compositional change of the human gut microbiome after these dietary restrictions were explored.

Our feasibility study displayed very high compliance despite the moderate side effects of the fasting intervention, suggesting that large-scale studies with two fasting intervention phases as well as additional inulin intake are feasible (Table 1). Inulin intake caused discomfort such as bloating, flatulence, and diarrhea and did not affect compliance. The provided vegetable juices were unpopular during the fasting interventions and some participants even expressed difficulty consuming the full amount of juice with the daily 300 kcal during the three days of fasting. As our study participants indicated that they would participate in further fasting interventions but would prefer vegetable broth to vegetable juices, we suggest testing different types of juices or using study designs with vegetable broth instead of juice. Not only could this increase compliance, but it could also lead to even clearer results, as He et al. reported in an intervention study of 16 participants that water-only fasting had a more profound and lasting effect on gut microbiome composition, while juice fasting had a relatively limited effect on the gut microbiome [47].

Our pilot study included six participants and was not powered to reveal significant changes in the gut microbial composition. Additional studies with larger numbers of participants will be needed to determine our observations. In general, results from the 16S rRNA gene analysis of the stool samples suggest that the variation in microbial composition was dominated by inter-individual variation. Although we did not observe significant changes within the gut microbial composition among the study participants, quantitative changes in the microbial composition suggest a decrease in alpha-diversity after fasting. Although some other studies rather suggest an increase in diversity measures [47,48], Gabel et al. reported that phylogenetic diversity remained unchanged in a study with an 8 h time restricted feeding intervention [49]. Interestingly, the fasting intervention with additional inulin intake displayed a slightly lower decrease in diversity measurements. These results hint that inulin consumption might mitigate the loss of diversity after a three-day juice fasting intervention.

We detected an increase in the *Akkermansia* genus in the gut microbiome of five of the six participants after the fasting intervention. Other studies demonstrate a similar increase in this genus in association with fasting periods [31]. *Akkermansia municiphila* has been associated with anti-inflammatory effects that are beneficial for inflammatory bowel diseases such as ulcerative colitis and has also been shown to positively influences metabolic health [31]. The relative abundance of the genera *Eubacterium retale* and *Fusicatenibacter* decreased after the fasting interventions. Both genera belong to the *Lachnospiraceae* family, which is part of the core gut microbiota [50], and both have been suggested to be associated with obesity [51]. After the fasting intervention with additional inulin intake, we detected a clear increase in the relative abundance of *Bifidobacterium*. This genus is a well-known commensal of the human gut and has been observed to be able to metabolize inulin and produce antimicrobial agents that protect the host from opportunistic pathogens. Interestingly, this increase in the relative abundance of *Bifidobacterium* appeared to be more pronounced among study participants who reported tolerating inulin well. As our study population is too small to draw firm conclusions, we suggest conducting studies with larger sample sizes to investigate possible correlations between inulin tolerability and efficacy, as this information may be relevant for predicting the outcomes of prebiotic treatments in the context of preventive medicine.

Our pilot study suggests that juice fasting intervention studies with two interventions consisting of three consecutive days of reduced caloric intake to a minimum of 300 kcal per day are feasible. In addition, the study provides a first indication of possible associations between inulin consumption during juice fasting and the microbial composition of the human gut suggesting a decrease in microbial diversity. A longer duration of juice fasting interventions or long-term intermittent fasting may display more profound effects on the gut microbiota. Although an additional inulin intake did not improve compliance in our study, 16S rRNA gene analysis suggests that inulin consumption reduces the loss of gut microbial diversity after a juice fasting intervention and leads to an increase in *Bifidobacterium* in participants who tolerated it well. As the side effects in our study were tolerable, our results suggest that prebiotic inulin can be integrated into a fasting protocol to investigate its effects on the composition of the human gut microbiome. Additional inulin consumption could be particularly beneficial for individuals with type 2 diabetes or metabolic syndrome, as the resulting SCFA may lead to health benefits such as improved glucose homeostasis, blood lipid profiles, and reduced body weight. Therefore, larger studies with more participants should be conducted, particularly to investigate the role of inulin in this matter.

## 5. Conclusions

In this study, we examined the feasibility of conducting a juice fasting intervention in combination with the consumption of the prebiotic inulin. This study design provides a targeted look at possible associations between inulin consumption during juice fasting and the composition of the human gut microbiome due to the greatly reduced amount of other dietary components in a more complex habitual diet. Our pilot study describes early tendencies in potentially expectable effects on the gut microbiome and provides a basis for further studies with a larger sample size to illuminate the health effects of juice fasting and inulin consumption and how these might be mediated by the gut microbiome.

## Figures and Tables

**Table 1:** Detailed information about study feasibility for each of the six participants. “yes” and “no” responses are color coded to differentiate favorable (light grey) from poor (dark grey) tolerance of the study design. Scoring answers are color coded with a gradient green scale from favorable (transparent/score = 1) to poor (green/score = 10) tolerance.

	P1	P2	P3	P4	P5	P6
Feasibility of the study design						
Did you find it difficult to give up solid food completely for three days?	no	no	yes	yes	yes	no
Was the daily amount (300 kcal) of vegetable juices sufficient for you to keep up the fast?	yes	yes	no	yes	yes	yes
Did you find it difficult to fast twice?	no	no	no	no	yes	no
Did you find the time between the two fasting periods sufficient?	yes	yes	yes	yes	yes	yes
Side effects						
Did you have side effects during the fasting periods?	no	yes	yes	yes	yes	yes
If yes, how much did these side effects interfere with your daily life? (Not at all 1, Very much 10)	1	1	4	10	7	5
Did you tolerate the inulin well?	yes	no	no	no	yes	yes
Adaptations for future studies						
Could you imagine giving up solid food completely for five days?	yes	yes	-	yes	no	yes
With tea only	yes	yes	-	no	no	-
With vegetable broth and tea only	yes	yes	yes	yes	yes	yes

## Data Availability

The data presented in this study are available on request from the corresponding author.

## Appendix A

**Table A1:** Details of the diet during the 5-day fasting intervention.

**Day of** **Intervention**	**Number of Calories Consumed**	**Diet Plan**
Day 1		Light, vegan diet:
	800 kcal	With vegetables, fruits, cereals, bread
		Without meat, fish, dairy products, added sugar, sweets, alcohol, and caffeine
Day 2		1 pkg. carrot juice (155 kcal/500 mL)
	300 kcal	1 pkg. sauerkraut juice (50 kcal/500 mL) 1/2 pkg. beetroot juice (105 kcal/250 mL)
Day 3		1/2 pkg. beetroot juice (105 kcal/250 mL)
	300 kcal	1 pkg. tomato juice (80 kcal/500 mL)
		1 pkg. mixed vegetable juice * (95 kcal/500 mL)
		1/2 pkg. sauerkraut juice (25 kcal/500 mL)
Day 4	300 kcal	1 pkg. tomato juice (80 kcal/500 mL) 1 pkg. carrot juice (155 kcal/500 mL)
		1 pkg. sauerkraut juice (50 kcal/500 mL)
Day 5	800 kcal	Light, vegan diet: With vegetables, fruits, cereals, bread
		Without meat, fish, dairy products, added sugar, sweets, alcohol, and caffeine

* Ingredients mixed vegetable juice: Tomato juice 67%, carrot juice 13%, sauerkraut juice 6%, celery juice 4%, beetroot juice 4%, cucumber juice 2%, paprika pulp 2%, onion juice, bean juice, dill juice, and herb sea salt (sea salt, herbs).

**Table A2:** Nutrient composition of the juices used in the study.

Type of Juice	Nutrient Composition
Tomato juice	0.50 g fat of which 0.10 g saturated fat,
	3.10 g carbohydrates of which 2.80 g sugar,
	0.50 g fiber, 1 g protein, 0.40 g salt, 0.25 sodium
Mixed vegetable juice	0.50 g fat of which 0.10 g saturated fat,
	3.50 g carbohydrates of which 3.30 g sugar, 0.60 g fiber, 0.90 g protein, 0 g salt
Beetroot juice	0.50 g fat of which 0.10 g saturated fat,
	9.20 g carbohydrates of which 9.20 g sugar,
	0.50 g fiber, 1 g protein, 0.13 g salt, 0.05 sodium
Sauerkraut juice	0.50 g fat of which 0.10 g saturated fat,
	1.50 g carbohydrates of which 1 g sugar, 0.50 g fiber, 1 g protein, 0.60 g salt, 0.60 sodium
	0.50 g fat of which 0.10 g saturated fat,
Carrot juice	6.80 g carbohydrates of which 6.80 g sugar,
	0.50 g fiber, 1 g protein, 0.13 g salt, 0.05 sodium
